# Carcinome cutané de Merkel: apport de la TEP-TDM au^18^FDG

**DOI:** 10.11604/pamj.2016.23.156.8571

**Published:** 2016-04-05

**Authors:** Guensi Amal, Taleb Sara, Cherkaoui Salhi Ghofrane, Ait Idir Malika, Houjami Majdouline, Sahraoui Souha, Benider Abdelatif, Touil Najoua, Benmoussa Ghita, Baroudi Zineb, Chikhaoui Nabil

**Affiliations:** 1 Service de Médecine Nucléaire, CHU Ibn Rochd, Rabat, Maroc; 2 Service d'Oncologie Radiothérapie, CHU Ibn Rochd, Rabat, Maroc; 3 Service de Radiologie des Urgences, CHU Ibn Rochd, Rabat, Maroc Taleb Sara, Service de Médecine Nucléaire, CHU Ibn Rochd, Rabat, Maroc

**Keywords:** Carcinome à cellules de Merckel, TEP-TDM, 18FDG, évolution, Cutaneous Merkel cell carcinoma, PET-CT, 18FDG, evolution

## Abstract

Le carcinome à cellules de Merkel (CCM) est une tumeur cutanée neuroendocrinerare d’évolution imprévisible et à grand potentiel métastatique. Ce néoplasme survient habituellement chez le sujet âgé au niveau des zones photo exposées. L'avidité constante du CCM au ^18^ fluorodésoxyglucose (FDG) justifie l'intérêt de la tomographie par émission de positon (TEP) au cours de cette pathologie. Toutefois, aucun consensus n'est établi à ce jour. Cette étude rapporte le cas d'une patiente de 25 ans suivie pour CCM métastatique, afin d'attirer l'attention sur cette tumeur particulière et d'illustrer l'intérêt de la TEP au 18 FDG dans la prise en charge de cette entité rare.

## Abstract (en)

Merkel cell carcinoma (MCC) is a rare neuroendocrine carcinoma of the skin with unpredictable evolution and high metastatic potential. This neoplasm usually occurs in the elderly on their sun-exposed areas. CCM constant greed for 18 fluorodeoxyglucose (FDG) defines the role of positron emission tomography (PET) in this pathology. However, no consensus has yet been reached. This study reports the case of a 25 year old woman treated for metastatic MCC, in order to draw attention to this particular tumor and to illustrate the role of 18 FDG PET in the management of this rare entity.

## Introduction

Le carcinome à cellules de Merkel (CCM) est une tumeur cutanée neuroendocrine rare et agressive, survenant fréquemment dans les zones photo exposées chez les personnes âgées. Plusieurs facteurs de risque sont décrits pour le CCM, bien qu'aucune étiologie ne soit retenue [1]. Le CCM est caractérisé par un potentiel évolutif rapide et un taux élevé de récurrence [2]. La tomographie par émission de positons couplée à la tomodensitométrie (TEP-TDM) au 18 F-Fluorodésoxyglucose (FDG) a été récemment proposée comme technique non invasive d’évaluation du CCM permettant une exploration à la fois morphologique et fonctionnelle. Les résultats des différentes études restent cependant variables ne permettant pas d’établir des recommandations précises [3]. Nous rapportons dans cette observation un cas de CCM métastatique, afin d'attirer l'attention sur cette tumeur particulière et d'illustrer comment l'examen TEP/TDM au FDG peut être contributif dans la prise en charge de cette pathologie.

## Patient et observation

**Figure 1:**
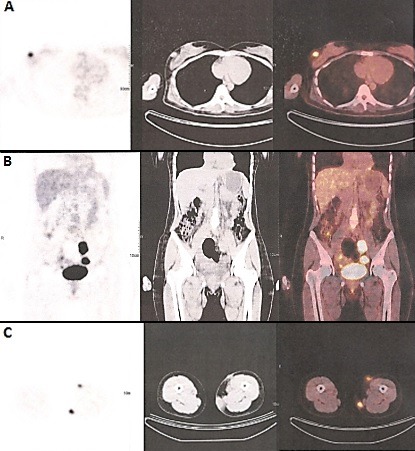
Bilan d'extension par TEP-TDM: (A) Nodule sous cutané du sein droit hypermétabolique (SUVmax à 6,55); (B) adénopathies hypermétaboliques iliaques internes bilatérales (SUVmax: à droite 9,29 et à gauche 22,31); (C) nodules cutanés hypermétaboliques de la face antérointerne et interne de la cuisse gauche (SUVmax respectif à 4,23 et 8,84)

La patiente E.A. âgée de 25 ans, célibataire et sans antécédents médicaux ou chirurgicaux, avait consulté pour une masse cutanée localisée au niveau du tiers inférieur de la jambe gauche qui s’était révélé bourgeonnante à l'examen clinique avec à des adénopathies inguinales homolatérales. La biopsie de cette masse a révélé un carcinome neuroendocrine cutané primitif à cellules de Merckel. Le bilan d'extension initial par imagerie morphologique n'a pas montré de localisations à distance. Le traitement chirurgical par exérèse large et curage ganglionnaire a été réalisé chez la patiente, complété par une radiothérapie externe sur le lit tumoral et le creux inguinal. L’évolution a été marquée une année plus tard par l'apparition de nouvelles localisations cutanées au niveau de la cuisse gauche, traitée par exérèse chirurgicale et radiothérapie centrée sur le lit tumoral. Une année après la fin de radiothérapie, la patiente a présenté une pesanteur pelvienne. L'examen clinique a montré une masse pelvienne, des nodules cutanés de la cuisse gauche et du sein droit.Le bilan morphologique a révélé une masse pelvienne au dépend de l'ovaire. La biopsie exérèse a confirmé la localisation ovarienne du carcinome à cellules de Merckel. Une TEP/TDM a été indiquée dans le cadre du bilan d'extension et a révélé: des adénopathies iliaques internes bilatérales hypermétaboliques, et des masses hypermétaboliques péritonéales et cutanées (au niveau de la cuisse gauche et du sein droit) (Figure 1). Devant ces résultats, une chimiothérapie à base cisplatine-etoposide a été indiquée. L'examen clinique après trois cures a objectivé une bonne réponse clinique.

**Figure 2:**
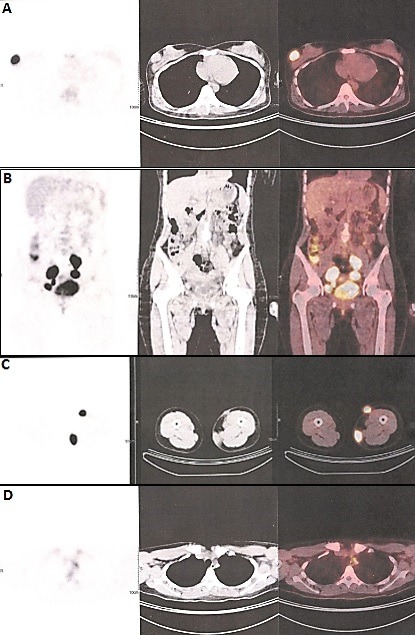
TEP-TDM d’évaluation thérapeutique: (A) une augmentation en volume et en intensité du nodule sous cutané du sein droit (SUVmax à 11,87 vs 6,55); (B) un magma d'adénopathies supravésical latéralisé à gauche et latérovésical droit (SUVmax respectif à 17,91 et 16,69) avec l'apparition d'adénopathies inguinales profondes; (C) une augmentation en volume et en intensité des nodules cutanés de la face antérointerne et interne de la cuisse gauche (SUVmax respectif à 12,64 et 15,37); (D) une adénopathie infracentimétrique latérotrachéale gauche de SUVmax à 3,58

Après cinq cures de chimiothérapie, l’évaluation TEP/TDM a conclu à un échec thérapeutique devant une augmentation en intensité et en étendue des foyers cutanés et l'apparition de nouvelles adénopathies hypermétabolisques médiastinales et pelviennes (Figure 2). Aussitôt, le protocole de chimiothérapie à base de cisplatine-etoposide a été remplacée par l'ifosfamide-doxorubicine avec cliniquement, une nette régression de la masse cutanée, toutefois les signes de mauvaise tolérance ont été constatés dès la première cure: aplasies médullaires à répétition malgré la réduction des doses et l'administration de facteurs de croissance médullaire. La TEP/TDM d’évaluation de la réponse à ce nouveau protocole de chimiothérapie après trois cures a montré: la persistance d'une adénopathie médiastinales hypermétabolique, la nette régression des nodules cutanés de la cuisse gauche et la disparition de la masse mammaire et des adénopathies pelviennes témoignant d'une rémission partielle de la maladie. Après la cinquième cure, l’évaluation clinique a noté la réapparition des nodules sous cutanés (cuisse gauche, sein droit). La patiente a été déclarée en échec thérapeutique et mise sous chimiothérapie orale à base d'Endoxan. La masse de cuisse gauche s'est compliquée par une gangrène de la cuisse puis un sepsis responsable du décès de la patiente.

## Discussion

Le CCM a été décrit pour la première fois par Toker en 1972 comme une tumeur trabéculaire du derme dérivée des cellules de Merkel [4]. Cette tumeur est restée relativement mal connue, de par sa rareté (0,3 pour 100 000 personnes/année aux Etats Unis d'Amérique [5]) ainsi que des critères histologiques disparates ou conflictuels. Cette incidence est toutefois en augmentation, selon les données disponibles [5]. L’étiologie du CCM reste inconnue à ce jour, cependant certains facteurs ont été incriminées: l’âge avancé, l'exposition solaire et l'immunodépression que la cause soit médicamenteuse (greffe ou maladie auto-immune) liée à une hématopathie ou au VIH [6]. Très récemment, il a été montré qu'un virus, désormais appelé Merkel cellpolyoma virus, était impliqué dans la pathogénie [3]. Dans le cas étudié la patiente ne présentait aucun de ces facteurs de risque et la sérologie viral en'a pas pu être réalisée. Le CCM est une tumeur agressive sur le plan locorégional et à distance: le risque de récidive locale est entre 27 à 52% et le risque de localisation secondaire est entre 18 à 52% selon les séries [1]. Du fait de son potentiel métastatique élevé, le CCM doit être gardé à l'esprit devant des métastases de primitif inconnu [7]. Les sites métastatiques les plus fréquents étant les ganglions lymphatiques, la peau, le poumon, le système nerveux central et l'os [8]. Trois cas de métastases ovariennes ont été rapportés dans la littérature [7] associées à des récidives locales ou des métastases cutanées. Dans notre cas, la patiente présentait aussi bien une extension ganglionnaire que des localisations secondaires: cutanées (cuisse gauche et sein droit), ovarienne et péritonéales. Le taux de mortalité du CCM est nettement plus élevé que celui du mélanome [3], avec une survie à cinq ans variant entre 30 et 64% [1]. La présentation clinique du CCM n'est pas spécifique. Typiquement, on le trouve en zone photo-exposée, surtout sur le visage et le cou (dans plus de 50% des cas); les localisations sur les extrémités; comme c'est le cas dans cette observation, sont plus rarement décrites [3]. Le diagnostic positif repose sur l'analyse histologique qui doit systématiquement être complétée par l'analyse du profil immuno histochimique avec recherche de l'expression de la cytokératine 20 (CK20), dont la positivité permet d'affirmer le diagnostic de CCM [1]. Un staging pré thérapeutique précis et rapide doit être effectué d'autant plus qu'il s'agit d'un facteur indépendant prédictif de survie [8]. Bien qu'aucun consensus ne soit établi quant au bilan d'extension à réaliser; un examen clinique complet de l'ensemble du tégument et de toutes les aires ganglionnaires est indispensable dans un premier temps. Une échographie ganglionnaire voire la ponction du ganglion sentinelle est recommandée par certaines équipes [2] en raison du caractère extrêmement lymphophile du CCM; associées à une tomodensitométrie (TDM) thoraco-abdominopelvienne pour déterminer les métastases à distance.

Au cas par cas, l'indication d'une imagerie en résonance magnétique (IRM) pourra être discutée en réunion de concertation pluridisciplinaire [1]. Le CCM est une tumeur avide de FDG, avec des valeurs moyennes de SUV max variables, comprises entre 4 et 13,6 [9] toutefois aucune association entre le SUV et le pronostic n'a été établis à ce jour. Les études qui ont évalué l'utilité de la TEP et de la TEP-TDM chez les patients atteints de CCM rapportant des résultats variables. Cependant, la puissance statistique de ces travaux reste limitée, étant donné la rareté de la pathologie et donc le faible effectif étudié en plus de la grande hétérogénéité des études de par les localisations variables de la maladie. S'ajoute aux facteurs en rapport avec la maladie (stade et expression de la maladie, localisations secondaires, thérapeutiques entamées…), l'hétérogénéité technique due à la diversité des méthodes utilisées (TEP seule ou TEP-TDM, activité injectée, le temps entre l'injection et l'acquisition des images) [9]. Les résultats restent néanmoins très encourageants: la sensibilité de la TEP-TDM dans l'exploration du CCM est de 90%; et la spécificité est supérieure à 98% [8]. Ainsi, le National Compréhensive Cancer Network (NCCN) justifie la réalisation de la TEP-TDM dès que l'extension est cliniquement décelable [10]. D'autres études récentes, préconisent sa réalisation chez tout patient atteint de CCM que ce soit lors du bilan initial, permettant de modifier le staging dans 33% des cas [11] ou lors des trois premières années du suivi, justifiant la modification de la prise en charge chez 37% des patients [9]. Il a également été démontré que la présence d'une maladie métabolique active sur les images TEP a été associée à une diminution de la survie. Des faux négatifs sont néanmoins rapportés pour les lésions de petite taille (inférieure à la résolution de la technique), avec une faible activité proliférative ou situées dans les sites de fixation physiologique du FDG. Les rares faux positifs rapportés sont dus à la confusion avec des lésions inflammatoires [9]. A ce jour, aucune étude comparant la TEP-TDM à d'autres techniques d'imagerie n'a été rapportée. Seuls Colgan et al. lors d'une étude rétrospective, ont décrété que la TEP seule est beaucoup plus sensible que la TDM dans l’évaluation de l'envahissement ganglionnaire chez les patients atteints de CCM [12]. De point de vu thérapeutique, il n'existe aucun consensus clairement établis concernant les modalités à adopter face à cette tumeur, toutefois, le traitement est avant tout chirurgical. La reconstruction cutanée privilégiera des techniques simples facilitant la surveillance ultérieure [1]. Si le ganglion sentinelle est positif, un curage ganglionnaire complémentaire est réalisé préférablement dans le même temps chirurgical. En complément de la chirurgie, une radiothérapie est systématiquement recommandée sur le site tumoral primitif, et sur l'aire ganglionnaire de drainage et semble diminuer le risque de récidive locorégionale et améliorer la survie globale [1]. Il est nécessaire de noter qu’étant donné le potentiel évolutif élevé du CCM, une prise en charge rapide est essentielle. En effet, un délai supérieur à 24 jours avant la radiothérapie est associée à un risque élevé de progression [2]. La chimiothérapie est indiquée uniquement en cas de métastases à distance, bien qu'il n'existe pas de données montrant une amélioration du pronostic en cas de maladie métastatique [12]. A ce stade de la maladie, une prise en charge palliative peut être discutée.

## Conclusion

La TEP-TDM au ^18^ FDG semble être un examen très utile dans la prise en charge des patients atteints de CCM que ce soit lors du bilan d'extensioninitial ou lors de l’évaluation de la réponse au traitement. Les données de la littérature restent néanmoins encore limitées notamment dans notre contexte: des études multicentriques sur de grands échantillons sont nécessaires pour évaluer avec précision l'apportde la TEP/TDM dans la prise en charge des patients avec un CCM dans le but d'optimiser leur prise en charge thérapeutique.
